# Gel shift analysis of the *empA *promoter region in *Vibrio anguillarum*

**DOI:** 10.1186/1471-2180-4-42

**Published:** 2004-10-29

**Authors:** Steven M Denkin, Pedja Sekaric, David R Nelson

**Affiliations:** 1 Department of Cell and Molecular Biology, University of Rhode Island, Kingston, RI 02881, USA; 2 Department of Molecular Microbiology and Immunology, Bloomberg School of Public Health, Johns Hopkins University, 615 N. Wolfe Street, Baltimore, MD 21205, USA; 3 Department of Medicine, University of Massachusetts Medical School, LRB 360D, 364 Plantation Street, Worcester, MA 01605, USA

## Abstract

**Background:**

The induction of metalloprotease encoded by *empA *in *Vibrio anguillarum *occurs at high cell density in salmon intestinal mucus. Previously we have shown that there are significant differences in *empA *expression in two strains of *V. anguillarum*, M93Sm and NB10. It is hypothesized that differences in *empA *regulation are due to differences in binding of regulatory elements.

**Results:**

Two strains of *V. anguillarum*, M93Sm and NB10, were examined and compared for the presence of DNA regulatory proteins that bind to and control the *empA *promoter region. Gel mobility shift assays, using a digoxigenin (DIG)-labeled oligomer containing a *lux *box-like element and the promoter for *empA*, were done to demonstrate the presence of a DNA-binding protein. Protein extracts from NB10 cells incubated in Luria Bertani broth + 2% NaCl (LB20), nine salts solution + 200 μg/ml mucus (NSSM), 3M (marine minimal medium), or NSS resulted in a gel mobility shift. No gel mobility shift was seen when protein extracts from either LB20- or NSSM-grown M93Sm cells were mixed with the DIG-labeled *empA *oligomer. The azocasein assay detected protease activity in all incubation conditions for NB10 culture supernatants. In contrast, protease activity was detected in M93Sm culture supernatants only when incubated in NSSM. Since the *luxR *homologue in *V. anguillarum*, *vanT*, has been cloned, sequenced, and shown to be required for protease activity, we wanted to determine if *vanT *mutants of NB10 exhibit the same gel shift observed in the wild-type. Site-directed mutagenesis was used to create *vanT *mutants in *V. anguillarum *M93Sm and NB10 to test whether VanT is involved with the gel mobility shift. Both *vanT *mutants, M02 and NB02, did not produce protease activity in any conditions. However, protein extracts from NB02 incubated in each condition still exhibited a gel shift when mixed with the DIG-labeled *empA *oligomer.

**Conclusions:**

The data demonstrate that protein extracts of *V. anguillarum *NB10 cells contain a protein that binds to a 50 bp oligomer containing the *empA *promoter-*lux *box-like region. NB10 cells express *empA *during stationary phase in all growth conditions. The DNA binding protein is not present in M93Sm extracts. M93Sm cells express protease activity only when incubated at high cell density in fish gastrointestinal mucus. The gel shift observed with NB10 cells is not due to VanT binding. The data also suggest that the DNA binding protein is responsible for the less restrictive expression of *empA *in NB10 compared to M93Sm.

## Background

*Vibrio anguillarum *is the causative agent of vibriosis, one of the major bacterial diseases affecting fish, bivalves, and crustaceans [1-3]. Vibriosis has been a major problem for the aquaculture industry around the world. Large economic losses due to this fish pathogen are sustained by the fish farming industry. Annual losses of cultured fish species in Japan alone exceed $30 million [1]. Vibriosis in fish is observed as a hemorrhagic septicemia. Infected fish display skin discoloration and erythema around the base of the fins, vent, and mouth. Necrotic lesions form in the abdominal muscle. Mortalities within affected fish farm stocks range from 30–100% [1,4,5].

Extracellular metalloproteases are important virulence factors for many pathogenic bacteria including *Vibrio cholerae *[6,7] and *Vibrio vulnificus *[8-10]. The EmpA metalloprotease genes of *V. anguillarum *strains NB10 and M93Sm have been cloned and sequenced by Milton et al [11] and Denkin and Nelson [12], respectively. The EmpA metalloprotease of *V. anguillarum *shares significant sequence homology with other known proteases. Although the role of the EmpA metalloprotease is not completely understood, we showed previously that *V. anguillarum empA *mutants are either avirulent or attenuated for virulence in Atlantic salmon depending on the route of inoculation [12]. It can be hypothesized that EmpA causes tissue damage during pathogenesis.

Recently, the *Vibrio harveyi luxR *(LuxR_Vh_) homologue in *V. anguillarum*, *vanT*, was cloned, sequenced, and shown to be required for protease activity [13]. LuxR_Vh _dependent regulation of luminescence in *V. harveyi *occurs at the promoter of the *lux *operon [14,15]. While the amino acid sequence of LuxR_Vh _in *V. harveyi *is not similar to the LuxR_Vf _protein in *V. fischeri *which also regulates luminescence [16], it is thought that LuxR_Vh _binds to the *lux *box and activates the expression of the luciferase genes. Since LuxR_Vh _functions as a transcriptional activator in *V. harveyi *and VanT is required for protease expression in *V. anguillarum*, we wanted to determine whether any proteins bind to the regulatory regions (e.g. the *lux *box-like element and promoter) immediately upstream of *empA*.

We have previously shown that *V. anguillarum *cells grow rapidly in Atlantic salmon intestinal mucus [17,18] and that *empA *is strongly induced when cells are incubated at high density in mucus [17]. Further, we have demonstrated that *empA *transcription is RpoS-dependent [12]. Our findings also suggest that quorum sensing and undescribed autoinducer(s) help to regulate the expression of *empA *in *V. anguillarum *[12]. Additionally, Milton et al [19] previously identified a *lux* box-like element about 70 bp upstream of the translational start of *empA*, which we subsequently showed to span the transcriptional start site [12]. In this study, we examine, compare, and contrast the expression of metalloprotease encoded by *empA *in two strains of *V. anguillarum *(M93Sm and NB10). Differences in *empA *expression were investigated with regards to various growth conditions, the presence or absence of DNA-binding proteins, and putative regulatory proteins.

## Results

### Gel shift analysis of the *empA *promoter

**Figure 1:**
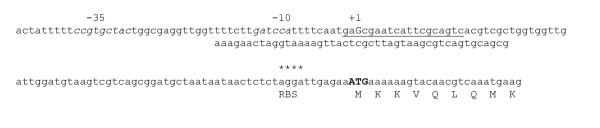
**Nucleotide sequence of the promoter region of *empA***. The 5'-coding sequence and immediate upstream/promoter sequence of *empA *is presented. The base-paired sequence indicates the annealed primers empA3W and empA3C (Table 2). The transcriptional start site is indicated by +1, the *rpoS*-dependent promoter regions are indicated as -10 and -35 with the bases in italics [12], and the *lux *box-like element [19] is underlined. Additionally, the start translation site (ATG) is in bold with the translated product below and the ribosome binding site (RBS) is marked above (*).

**Figure 2:**
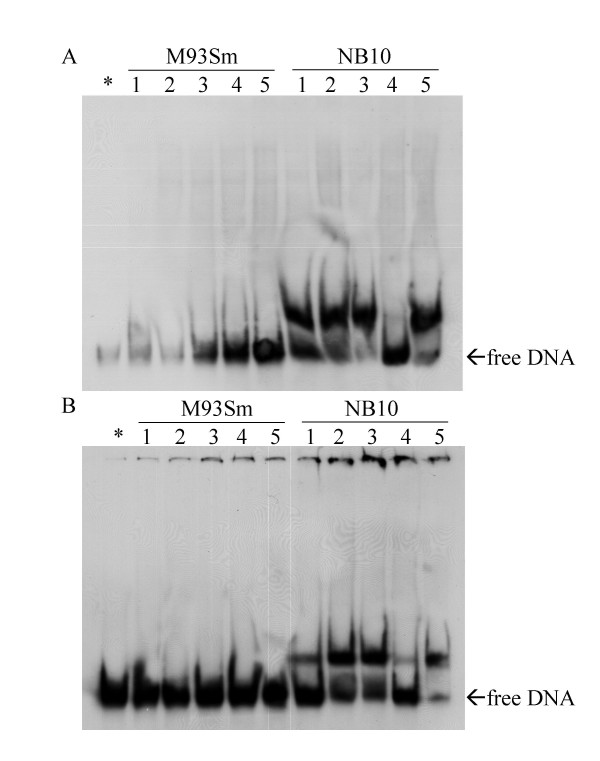
**Gel shift analysis using a DIG-labeled 50 bp oligomer (0.8 ng/lane) containing the *empA *promoter region and putative *lux *box. **Protein extracts were prepared from *V. anguillarum *M93Sm (left) and NB10 (right) incubated in (A) LB20 or (B) NSSM and added to DIG-labeled DNA. DIG-labeled DNA alone was added to the lane marked with an asterisk (*). Lane 1, 5 μg of protein extract from cells at time 0 h; lane 2, 5 μg protein extract from cells at time 3 h; lane 3, 7.5 μg protein extract from cells at time 3 h; lane 4, 5 μg protein extract from cells at time 3 h plus the unlabeled *lux *box-*empA *promoter containing oligomer; and lane 5, 5 μg protein extract from cells at time 3 h plus non-competitive oct2A (from *E. coli*) unlabeled 39 bp oligomer. The data presented are representative of three replicate experiments.

**Figure 3:**
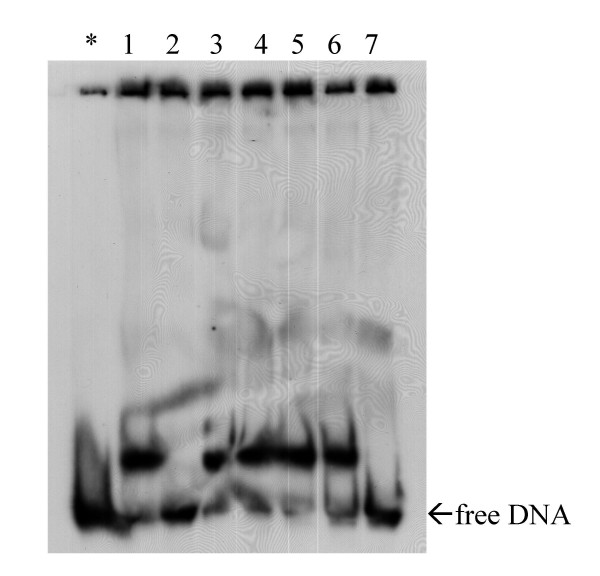
**Gel shift analysis of proteins from NB10 cells incubated in 3M and NSS. **Cells grown overnight in LB20 were washed twice in NSS and resuspended at 2 × 10^9 ^cfu/ml in the appropriate medium. Protein extract (5 μg) from cells incubated for 3 h in each condition was added to the gel shift reaction with the DIG-labeled oligomer. DIG-labeled DNA alone was added to the lane marked with an asterisk (*). Reactions containing protein extracts prepared from each condition was loaded onto the gel: lane 1, NSSM; lane 2, NSSM + unlabeled oligomer; lane 3, LB20; lane 4, 3M; lane 5, NSS; lane 6, exponential phase cells in LB20; and lane 7, M93Sm in NSSM. The data presented are representative of three replicate experiments.

The possibility of DNA-binding proteins interacting with regulatory sequences of *empA*, were examined using a digoxigenin (DIG) labeled oligomer containing the *lux *box-like element and *empA *promoter region. The location of double stranded oligomer relative to the promoter and coding sequences is shown in Fig. 1. Protein extracts from *V. anguillarum *M93Sm and NB10 cells incubated in LB20 and NSSM were mixed with the DIG-labeled oligomer (Fig. 2). NB10 and M93Sm cells were incubated in LB20 and NSSM for 3 h and protein extracts from cells at 0 and 3 h were prepared. DNA binding was observed in protein extracts from NB10 incubated in LB20 and NSSM (Fig. 2A and 2B) at both 0 and 3 h. A 3.7-fold increase (from T = 0 h) in the amount of DNA binding was observed by 3 h in extracts from NSSM-grown NB10 cells (Fig. 2B, NB10 lane 2). An additional 1.4-fold increase in DNA binding was observed when the amount of protein was increased from 5 μg to 7.5 μg (Fig. 2B, NB10 lane 3). Although only a minor change in the amount of DNA binding (1.4-fold increase) was detectable by 3 h in NB10 extract from LB20-grown cells, a 4.7-fold decrease (from T = 0 h) in free DIG-labeled oligomer was observed (Fig. 2A, NB10 lane 2). In addition, a 8.9-fold decrease in free DIG-labeled oligomer was detected when 7.5 μg protein was added (Fig. 2A, NB10 lane 3). Specificity of protein binding to the DIG-labeled oligomer was tested by competitive inhibition of binding by the addition of excess amounts of an identical unlabeled oligomer. When excess unlabeled *lux *box-*empA *promoter oligomer was added to protein extracts from LB20- and NSSM-grown NB10 cells binding to the DIG-labeled oligomer was abolished. Further, the addition of unlabeled oct2A (a non-competitive oligomer) to protein extracts did not affect the gel mobility shift of the DIG-labeled oligomer caused by protein extracts from NB10 cells incubated either in LB20 or NSSM. In contrast to protein extracts from NB10 cells grown in LB20 or NSSM causing a gel shift, protein extracts from M93Sm cells incubated in either condition did not show any binding to the DIG-labeled oligomer (Fig. 2A and 2B). Even when 7.5 μg of protein was used, a band shift was not observed. In a separate experiment, protein extracts from NB10 cells incubated in 3M or starved in NSS also caused a similar gel mobility shift for the DIG-labeled oligomer (Fig. 3). Additionally, even when 5 μg of protein extract from exponentially growing NB10 cells in LB20 was added to the DIG-labeled oligomer, a band shift was observed. Further, in this gel shift experiment, M93Sm protein extract was added as a negative control to the labeled DIG-labeled oligomer and no shift was observed.

### Protease activity in LB20, NSSM, 3M, and NSS

**Figure 4:**
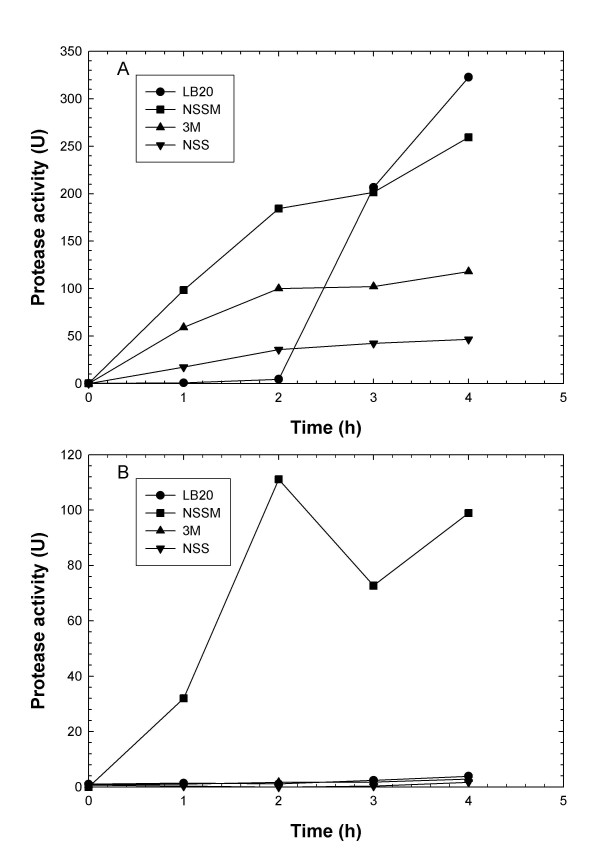
**Induction of protease activity in *V. anguillarum *NB10 (A) and M93Sm (B) cells incubated in LB20 (●), NSSM (■), 3M (▲) or NSS (▼). **Cells were grown for 16 h in LB20 at 27°C, harvested by centrifugation, washed twice in NSS, and resuspended at 2 × 10^9 ^CFU/ml in each condition. Samples were taken at the indicated times and cell-free supernatant was tested for protease activity using the azocasein assay. Both of the experiments shown represent a single experiment, although each experiment was repeated at least three times with similar results.

Expression of *empA *in NB10 occurs in both LB20 and NSSM, while M93Sm exhibits *empA *expression only in NSSM [12]. Since a gel mobility shift was observed in NB10 protein extracts in all conditions of growth and during starvation, these cells were tested for protease activity in 3M and NSS in addition to LB20 and NSSM (Fig. 4). Protease activity was detected in NB10 cells after 1 and 3 h of incubation in NSSM or LB20, respectively (Fig. 4A). Maximum protease activity was observed at 4 h for both conditions of incubation. In addition, protease activity was observed in NB10 cells incubated in 3M or starved in NSS after 1 h and continued to increase during the next hour of the 4 h experiment. Throughout the incubation, NB10 cells in 3M produced 3–4 fold greater amounts of protease activity than cells in NSS. The amount of protease measured in NSS at 4 h was only 14 and 17% of the amount produced in LB20 and NSSM, respectively. In contrast, protease activity was observed in M93Sm cells only when incubated in NSSM (Fig. 4B). M93Sm cells did not produce protease activity in any of the other conditions examined.

### Effects of *vanT *mutations on protease activity and gel mobility shift

**Figure 5:**
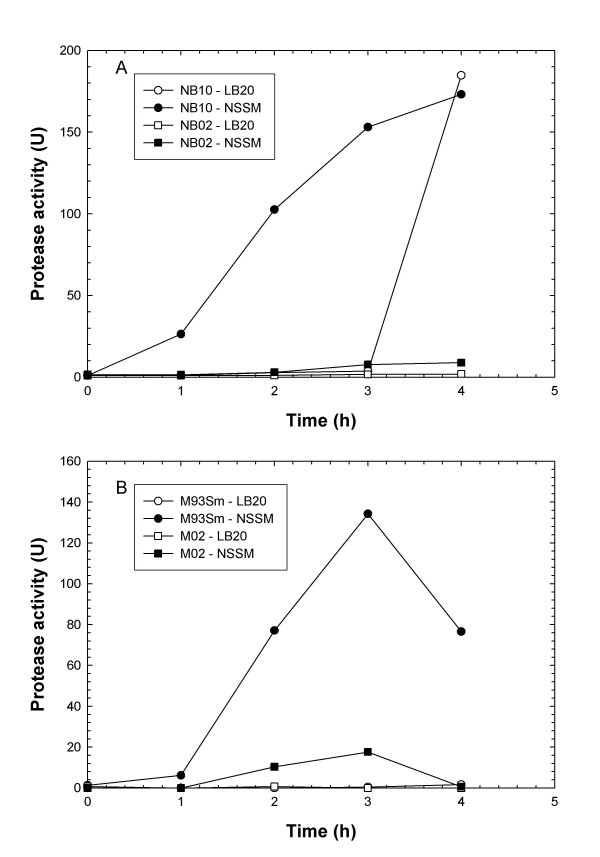
**Induction of protease activity in *V. anguillarum *wild type (circles) and *vanT *mutant strains (squares). **Cells were grown for 16 h in LB20, harvested by centrifugation, washed twice in NSS, and resuspended at 2 × 10^9 ^CFU/ml in either LB20 (open symbols) or NSSM (solid symbols). (A) NB10 and *vanT *mutant, NB02. (B) M93Sm and *vanT *mutant, M02. Both of the experiments shown represent a single experiment, although each experiment was repeated at least three times with similar results.

Since *vanT *of *V. anguillarum *NB10 was cloned, sequenced and shown to be required for protease activity [13], *vanT *mutants of both M93Sm and NB10 were created by site directed mutagenesis and tested for protease activity in LB20 and NSSM. When the mutants (NB02 and M02) were incubated in NSSM for 3 h, a small amount of protease activity was detected (5 and 13% of the wild-type levels, respectively) (Fig. 5). No protease activity was observed in M93Sm, M02, or NB02 incubated in LB20.

**Figure 6:**
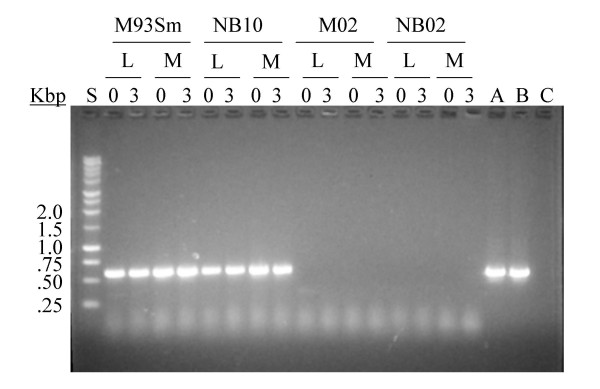
**RT-PCR analysis of *vanT *expression in wild-type (M93Sm and NB10) and *vanT *mutant (M02 and NB02) strains of *V. anguillarum*.** Cells were grown overnight in LB20, washed twice in NSS, and resuspended in either LB20 or NSSM at 2 × 10^9 ^CFU/ml. RNA from LB20- (L) and NSSM- (M) grown cells was prepared and used in RT-PCR reactions. RNA was extracted from cells at 0 h and 3 h after resuspension. As a positive control, PCR was performed on DNA from M93Sm (lane A) and NB10 (lane B). As a negative control, RT was omitted from the reaction containing RNA samples; a representative result is shown in lane C. The PCR and RT-PCR products were visualized on a 1% agarose TAE gel containing ethidium bromide. Molecular weight standard (indicated in kilobase pairs) are in lane S. The data presented are representative of three replicate experiments.

The *vanT *mutant strains (M02 and NB02) and their parental wild-types (M93Sm and NB10, respectively) were also examined for *vanT *transcription by RT-PCR (Fig. 6). Neither *vanT *mutant produced *vanT *transcripts. Interestingly, both wild-type strains produced *vanT *mRNA in both LB20 and NSSM.

**Figure 7:**
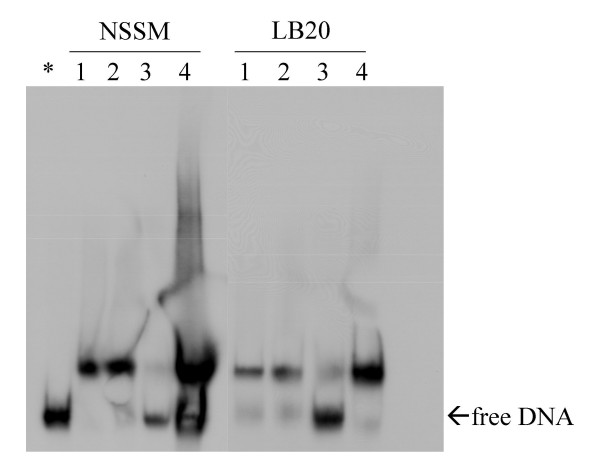
**Gel shift analysis of the *V. anguillarum vanT *mutant, NB02 in NSSM and LB20. **The same labeled oligomer used in Figures 1 and 2 was added to protein extracts from NB02 cells incubated in NSSM and LB20. DIG-labeled DNA alone was added to the lane marked with an asterisk (*). Lane 1, 5 μg of protein extract from cells at time 0 h; lane 2, 5 μg protein extract from cells at time 3 h; lane 3, 5 μg protein extract from cells at time 3 h plus unlabeled *lux *box-*empA *promoter; and lane 4, 5 μg protein extract from cells at time 3 h plus the oct2A (from *E. coli*) unlabeled oligomer. The data presented are representative of three replicate experiments.

To determine if VanT was binding to the *lux *box-*empA *promoter, protein extracts from NB02 were tested in the gel shift assay (Fig. 7). Protein extracts from NB02 cells incubated in NSSM and LB20 exhibited binding to the DIG-labeled oligomer similar to that observed with protein extracts from wild-type NB10 cells (compare to Fig. 2). These data show that VanT was not responsible for the gel mobility shift of the *lux *box – *empA *promoter oligomer.

## Discussion

It has been demonstrated that the transcriptional activator, LuxR_Vf_, in *Vibrio fischeri*, activates expression of the *lux *operon via interaction with the *lux *box [15,16,20,21]. Although there is incomplete understanding of LuxR_Vf _interaction with the *lux *box, good evidence is available that shows a specific *lux *box sequence required for efficient LuxR_Vf _binding [21]. LuxR_Vh _from *V. harveyi *and LuxR_Vf _from *V. fischeri *are not the same transcriptional activator proteins [22]. These proteins differ in their activation of the *lux *operon. In *V. fischeri*, LuxR_Vf _interacts with an acylhomoserine lactone autoinducer and then binds to the *lux *box to activate transcription of the *lux *genes [23]. In *V. harveyi*, the regulation of luminescence occurs via a phosphorelay signal transduction system, which involves LuxR_Vh _binding to the *lux *box independent of an autoinducer [24]. Additionally, a central response regulator, LuxO, has been shown to regulate the expression of *luxR *[24] in *V. harveyi*. Recently, the LuxR_Vh _homologue of *V. fischeri *was identified as LitR [25]. However, it is not known if *V. fischeri *uses a phosphorelay cascade to control LitR interaction with the *lux *operon.

In this study, two strains of *V. anguillarum *(NB10 and M93Sm) were examined for a *empA *promoter region binding protein. In all conditions, incubation with NB10 protein extracts resulted in a gel mobility shift to the DIG-labeled oligomer. In contrast, M93Sm extracts did not show any protein binding to the labeled DNA in any conditions tested. This was interesting because NB10 protease production occurs in all conditions, while M93Sm protease activity is detected only when the cells are incubated in mucus. Recently, VanT, the LuxR_Vh _homolog in *V. anguillarum*, was identified by Milton et al [13] and shown to positively regulate EmpA metalloprotease and biofilm production. We created *vanT *mutants in both M93Sm and NB10 and tested them for *vanT *expression, protease activity, and DNA binding. These two mutants, M02 and NB02, did not produce detectable *vanT *mRNA. However, both exhibited low levels of protease activity in mucus - about 5% and 13% of wild type activity in mucus, respectively. For NB02, no protease activity in LB20 was observed. These results demonstrate that *vanT *is required for protease activity in a non-mucus containing medium (*V. anguillarum *NB10 in LB20), confirming the result reported by Milton et al [13]. Our results also show that in NSSM, *vanT *mutants still exhibit some protease activity, but VanT is required for full protease activity in both M02 and NB02 cells.

We tested a *vanT *mutant of *V. anguillarum *for the possibility of VanT binding to and activation of *empA *expression. Since the *V. anguillarum *NB10 strain expresses *empA *during stationary phase in all conditions tested and protein extracts from these conditions show DNA binding to the *empA *promoter region, it was hypothesized that VanT binds to this region. The *vanT *mutant, NB02, was analyzed for gel shift using protein extracts from cells incubated in LB20 and NSSM. Protein extracts from NB02 cells incubated in each condition still caused a band shift. This result suggests that VanT, the LuxR_Vh _homolog in *V. anguillarum*, either does not bind to the *empA *promoter region containing the *lux *box-like element or perhaps VanT binds to a second protein that binds to this DNA sequence. The former possibility is more likely since the gel shift observed in NB02 is identical to that observed in NB10 extracts. Further, since the M93Sm *vanT *mutant, M02 still exhibits about 13% of the wild type protease activity in NSSM and does not exhibit protein binding in the gel shift analysis, it is probable that VanT does not bind to the *empA *promoter-*lux *box region. However, Croxatto et al. [26] demonstrated recently that purified VanT protein binds to the *V. anguillarum *NB10 promoter regions for *empA*, *vanOU*, and *vanT*. Examination of their data shows that VanT binds more strongly to the promoters for *vanOU *and *vanT *(binding observed with 0.3 μg purified VanT per 2 ng DIG-labeled DNA) than to the *empA *promoter (binding observed with 2.5 μg VanT per 2 ng DIG-labeled DNA). It should be noted that in our study only 5 μg of total protein extract was used in the gel shift analysis. That binding to the *empA *promoter is still observed in *vanT *mutants strongly suggests that an unidentified protein binds to the promoter-*lux *box region.

Previously, we have shown that *empA *in *V. anguillarum *M93Sm is positively regulated by at least two components of salmon gastrointestinal mucus [17]. In this study we show that NB10 cells exhibit protease activity under all nutritional conditions including starvation in NSS when at high density, whereas M93Sm only induces protease activity in mucus. RpoS is required for expression of *empA *in both M93Sm and NB10 [12]. This was demonstrated by the observation that *rpoS *mutants of M93Sm (M03) and NB10 (NB03) exhibit no protease activity when cells are incubated in either LB20 or mucus during stationary phase. Extracellular factors present in conditioned media showed both positive and negative effects on *empA *expression [12]. LB20 conditioned supernatants from M93Sm cause a more rapid and increased production of protease activity (in NB10 and M93Sm cells). However, conditioned LB20 from the *luxS *mutant, M01added to wild-type cells results in a more rapid induction of protease activity, which suggests that AI-2 may act as a negative regulator of *empA*.

In this study we confirmed that VanT is required for *empA *expression in NB10 when incubated in LB20; however, when NB10 or M93Sm cells are incubated in NSSM, a small percentage of protease activity remains in the *vanT *mutants. Our data support the observation by Croxatto et al [13] that VanT positively regulates *empA *expression. Further, the role of VanT as a transcriptional activator of *empA *via the *lux *box-like element has not been established. Our data show that VanT does not bind to the *empA *promoter *lux *box-like region, but is required for maximum protease activity. In addition, we show that in NB10 an unknown protein binds to the *empA *promoter-*lux *box region that may increase protease activity in all conditions. In contrast, M93Sm protein binding to this region is not observed and protease activity is detected only when cells are incubated in mucus. In conclusion, the expression of the *empA *virulence gene in *V. anguillarum *is regulated transcriptionally by the alternative σ-factor RpoS (σ^S^) and the transcriptional activator VanT (a LuxR_Vh _homologue). Additionally, an unknown DNA binding protein binds specifically to the *empA *promoter *lux *box-like region in NB10 cells, but not in M93Sm cells. We hypothesize that this protein permits *empA *expression in the absence of the inducing factors in fish gastrointestinal mucus. A better understanding of how and why these two wild-type strains of *V. anguillarum *exhibit different levels of *empA *expression will further reveal how virulence genes are regulated in this fish pathogen.

## Conclusions

Differences in *empA *expression between the two strains of *V. anguillarum*, M93Sm and NB10, correspond with the production of an unknown regulatory protein that binds to a 50 bp oligomer identical to the promoter-*lux *box region of *empA*. For NB10, protein binding to the oligomer correlated with the expression of *empA *in all incubation conditions. However, M93Sm, which expresses *empA *only in mucus, did not exhibit protein binding in this condition. Additionally, a null mutation in *vanT *(*luxR *homologue) results in severely decreased or no *empA *expression, but does not abolish protein binding (in NB10) to the oligomer. Our data suggest that while *empA *expression in both *V. anguillarum *M93Sm and NB10 is dependent upon RpoS and VanT, the two strains differ in production of an unknown protein that binds to the promoter-*lux *box-like region of *empA*. We also hypothesize that the presence of this DNA binding protein permits NB10 cells to express *empA *in the absence of fish gastrointestinal mucus.

## Methods

### Bacterial strains, plasmids, and growth conditions

All bacterial strains and plasmids used in this report are listed in Table 1. *V. anguillarum *strains were routinely grown in Luria-Bertani broth + 2% NaCl (LB20) [18,27] supplemented with the appropriate antibiotic, on a rotary shaker at 27°C. *Escherichia coli *SM10 was grown in LB10 (LB + 1% NaCl) on a rotary shaker at 37°C. Experimental media included: LB20, 3M (marine minimal media) [18,28], nine salt solution (NSS, a carbon-, nitrogen-, and phosphorus-free salt solution) and NSS plus 200 μg mucus protein/ml (NSSM) [18]. Gastrointestinal mucus was harvested from Atlantic salmon as previously described by Garcia et al. [18]. Overnight cultures of *V. anguillarum *were grown in LB20, centrifuged (9,000 × *g*, 10 min), and pelleted cells washed twice with NSS [18]. Washed cells were resuspended to appropriate cell densities in experimental media. Specific conditions are described in the text for each experiment. Cell densities were determined by serial dilution and plating on LB20 agar plates or by measuring optical density at 600 nm (OD_600_). Antibiotics were used at the following concentrations: streptomycin (Sm), 200 μg/ml and chloramphenicol (Cm), 5 μg/ml.

### Bacterial matings

Plasmids were introduced into *V. anguillarum *M93Sm and NB10 from *E. coli *SM10 by conjugation using the procedure described by Milton et al. [11,29,30]. Briefly, overnight cultures of *V. anguillarum *M93Sm or NB10 and *E. coli *SM10 containing the pNQ705-1 *vanT *clone, pNQVanT, were prepared and mixed using ratios of 1:1 or 3:1 (recipient: donor) in NSS plus 10 mM MgSO_4_. The cell suspension was vacuum filtered onto a 0.22 μm nylon membrane, which was placed on an LB15 (LB + 1.5% NaCl) agar plate and allowed to incubate overnight at 27°C. Following incubation, the cells were removed from the filter by vigorous mixing in NSS plus 10 mM MgSO_4_. The cell suspension (100 μl) was plated on LB20 Sm^200 ^Cm^5 ^(for M93Sm mutants) or on TCBS Cm^5 ^(for NB10 mutants) and allowed to incubate at 27°C until *V. anguillarum *colonies were observed (usually 16–24 h).

### Site-directed mutagenesis of *vanT*

Site-directed mutagenesis was used to create gene interruptions within the structural gene of *vanT*. Primers (Table 2) were generated based on the *vanT *sequence for *V. anguillarum *NB10 (accession # AF457643). A 320 bp region from *vanT *was PCR amplified using Qiagen *Taq *DNA polymerase and cloned into the suicide vector, pNQ705, using *SacI *and *XbaI *restriction endonucleases to yield pNQVanT. The presence of the 320 bp *empA*-derived insert was confirmed by both PCR amplification and restriction analysis using *SacI *and *XbaI*. The mobilizable suicide vector, pNQVanT, was transferred into *V. anguillarum *by conjugation with *E. coli *SM10. *E. coli *SM10 contains the λ *pir *protein that is required for replication of pNQ705. Chloramphenicol resistant colonies were selected and screened for insertion within *vanT*. PCR and Southern blot analysis were used to confirm the incorporation of pNQVanT. For PCR analysis, a primer described previously by Milton et al. [11] complementary to the pNQ705 vector was utilized (Table 2). The forward primer, SD *vanT*-Forward (Table 2) is complementary to a region upstream of the insertion. PCR products were analyzed by electrophoresis through a 1.0 % agarose gel in Tris-acetate EDTA (TAE) [31] buffer containing 0.2 μg/ml ethidium bromide. The gene was interrupted within the 320 bp region of *vanT *rendering the mutants resistant to chloramphenicol at 5 μg/ml. The resulting *V. anguillarum vanT *mutants were designated M02 (derived from M93Sm) and NB02 (derived from NB10) (Table 1).

### Gel mobility shift assay

Protein extracts were prepared from 2 ml of cells using the Sigma CelLytic™ B bacterial cell lysis/extraction reagent containing 0.2 mg/ml lysozyme, 1 mM DTT, 2 mM EDTA, and 1 mM phenylmethylsulfonyl fluoride (PMSF). Briefly, the cells were pelleted from the experimental conditions by centrifugation at 9,000 × g for 10 min, 4°C. In addition, cells were lysed by multiple freeze-thaw cycles in crushed dry ice or in liquid nitrogen and then heated at 37°C. Insoluble cell debris was centrifuged at 12,000 × g for 10 min, 4°C. Protein concentration was determined using the Bradford assay (Bio-Rad).

The digoxigenin (DIG) gel shift kit for 3'-end labeling of oligonucleotides (Roche Applied Science, Indianapolis, IN) was used for protein-DNA binding assays. The 50 bp oligomers (Table 2) used contain the *empA *promoter region and putative *lux *box. These oligomers were synthesized and purified by HPLC (Integrated DNA Technologies, Coralville, IA). The oligomers were annealed, labeled, and used in the gel shift reactions according to the manufacturer's instructions (Roche). An 8% native polyacrylamide gel in 0.25 × TBE (Tris-borate-EDTA buffer) was prepared and used for electrophoresis (in 0.5 × TBE) of the gel shift reactions. Blotting was performed using a Biorad electro-blotting system (model Trans blot) according to the manufacturer's instructions. Chemiluminescence detection of DIG-labeled DNA-protein complexes on the nylon membranes was detected using Hyperfilm ECL (Amersham Pharmacia). Gel mobility shift fluorograms were examined quantitatively using a densitometer (Molecular Dynamics, Personal Densitometer, SI). All gel mobility shift experiments were repeated three times and the data presented are representative examples of each experiment.

### Detection and quantification of protease activity

Culture supernatants were assayed for proteolytic activity using our previously described modification of the method by Windle and Kelleher [17,32]. Briefly, culture supernatant was incubated with azocasein (5 mg/ml) dissolved in Tris-HCl (50 mM, pH 8.0) containing 0.04% NaN_3_. Culture supernatant was prepared by centrifuging 1 ml of cells at 12,000 × *g *(10 min, 20°C). Supernatant was removed and filtered through a 0.22 μm pore size cellulose-acetate filter. Filtered supernatant (100 μl) was incubated at 30°C with 100 μl of azocasein solution. Incubation time of 30 min was sufficient for assays of supernatants from cell suspensions of ≥ 5 × 10^8 ^cells/ml. Reactions were terminated by addition of trichloroacetic acid (TCA) (10% wt/vol) to a final concentration of 6.7% (wt/vol). The mixture was allowed to stand for 1 to 2 min and centrifuged (12,000 × *g*, 4 min) to remove unreacted azocasein, and supernatant containing azopeptides was suspended in 700 μl of 525 mM NaOH [32]. Absorbance of the azopeptide supernatant was measured at 442 nm using a Pharmacia Ultrospec 4000 spectrophotometer. A blank control was prepared by boiling *V. anguillarum *M93Sm supernatant (100°C, 10 min). TCA (final concentration 10%) was added to the blank control supernatant immediately after the addition of azocasein. The mucus used was also boiled (10 min) to destroy any inherent protease activity. Protease activity units were calculated using the following equation: 1 protease activity unit = [1000 (OD_442_)/CFU] × (1 × 10^9^)] [17].

### RNA isolation and RT-PCR conditions

Total RNA was isolated from *V. anguillarum *cells as previously described [12] using the RNeasy purification kit (QIAGEN). Reverse transcriptase (RT) reactions were performed using the OneStep RT-PCR system from QIAGEN. Prior to the RT reaction, RNA samples (2 μg) were treated with RQ1 (RNase-free) DNase (1 U/μl) according to the manufacturer's specifications (Promega, Madison, WI.). Primers used for RT-PCR reactions were *vanT*-F2 and *vanT*-R2 (Table 2). PCR amplification without the addition of RT was performed on an equal amount of RNA to demonstrate the absence of DNA. Amplification products were analyzed by agarose gel electrophoresis and sizes were determined using Kodak digital imaging software (Kodak 1D image analysis software, version 3.0.2; Eastman Kodak Co., Rochester, N. Y.).

### DNA sequencing

DNA sequencing was performed by the University of Rhode Island Genomics and Sequencing Center (Kingston, RI). Sequencing was performed on a Beckman-Coulter CEQ 8000. The Dye Terminator Cycle Sequencing (DTCS) quick start kit was used for the sequence reactions that were prepared according to the manufacturer's specifications and run in a thermal cycling program. DNA samples were mixed with the appropriate primer (Table 2) and then submitted for sequencing.

## Authors' contributions

S.M.D. carried out the experimental part of the study and drafted the manuscript. D.R.N. conceived of the study, participated in its design and coordination, and edited the manuscript. P.S. participated in the experiment design and assisted with gel shift analysis. All authors have read and approved the final manuscript.

## Figures and Tables

**Table 1:** Strains and plasmids used in this study

Strain or plasmid	Genotype and description	Source or reference
Strains		
*Vibrio anguillarum*		
M93Sm	Spontaneous Sm^R ^mutant of WT M93 (serotype J-O-1)	Denkin & Nelson [17]
M02	Sm^R ^Cm^R^; M93Sm *vanT *mutant	This study
NB10	WT (serotype O1)	Milton et al. [11]
NB02	Cm^R^; NB10 *vanT *mutant	This study
*Escherichia coli*		
SM10	*thi thr leu tonA lacY supE recA*	Milton et al. [11]
	RP4-2-Tc::Mu::Km (λ pir)	
Plasmids		
pNQ705-1	Cm^R^; suicide vector with R6K origin	Milton et al. [11]
pNQVanT	Cm^R^; 320 bp *vanT *in pNQ705-1	This study

**Table 2:** Oligomers used in this study

Oligomer	Sequence^a^
pNQ705-R	5'-GCGTAACGGCAAAAGCACCGCCGGACATCA-3'
SD*vanT*-F	5'-GGTGAGCTCCTGATGGAAATCGCTCTTGAAG-3'
SD*vanT*-R	5'-GGTTCTAGAGTGGCCACACTTCTTCGCG-3'
*vanT*-F2	5'-GGAAACATCGATAGAAAAACG-3'
*vanT*-R2	5'-GATGCAGAGCATGCTGAGG-3'
*empA*3W	5'-TGGTTTTCTTGATCCATTTTCAATGAGCGAATCATTCGCAGTCACGTCGC-3'
*empA*3C	5'-ACCAGCGACGTGACTGCGAATGATTCGCTCATTGAAAATGGATCAAGAAA-3'

^a ^Restriction sites *SacI *(GAGCTC) and *XbaI *(TCTAGA) are underlined.
